# Fibroid Tumor of Uterus Successfully Treated by Hypodermic Injections of Ergotine

**Published:** 1878-06

**Authors:** M. G. Sloan

**Affiliations:** Charlotte, Iowa


### Clinical Reports.

### NOTES FROM PRIVATE PRACTICE.

*Fibroid Tumor of Uterus successfully treated by Hypodermic Injections of Ergotine.*

On Nov. 25th, 1877, I was called to see Mrs. L., aged about 47. On inquiry, I found that she had been subject to an almost constant flow of blood from the vagina, for nearly two months past. She was so much exhausted by this as to be obliged to remain in bed. Defecation was performed with great difficulty, and the fæces were flattened. She also complained of various obscure pains over the region of the uterus and left ovary. She had been treated during this time for "change of life," and had been gradually getting worse.

I found the cervix in a normal position and apparently healthy. A body, which I at first supposed to be the uterus, occupied the posterior part of the pelvis. This body seemed to be of about the size of the clenched fist of an ordinary sized man, was immovable, and joined the cervix at about a right angle.

A rectal examination showed that the gut was almost occluded by this tumor. Being in doubt whether I had to deal with a retroflexed and hypertrophied uterus, or a fibroid tumor of the same, I introduced the uterine probe, which showed that the organ was in normal position, and that its cavity was slightly enlarged. The diagnosis then could admit of no doubt. Here was a uterine fibroid, which was already encroaching dangerously on the rectum, and was evidently gradually increasing in size, as was shown by the fact that defecation was performed with increasing difficulty. What was to be done? I had read many conflicting opinions as to the efficacy of hypodermic injections of ergotine in such cases, and as the preponderance of evidence seemed to be favorable, I determined to give the remedy a fair trial.

Without going into a detailed history of the case, it is sufficient to state that under this treatment, combined with drachm doses of fluid extract of ergot by the mouth three times daily, the tumor gradually became smaller, the hemorrhage at the same time diminishing, until at the end of one month (when treatment was discontinued), the hemorrhage had entirely ceased, the bowels were moved without difficulty, and the tumor was reduced to about the size of a small hickory nut. I am aware that the above stated result seems almost incredible, and yet "facts are stubborn things."

The injections were mainly made in the arm, and were repeated sometimes every day and sometime every other day.

The treatment was discontinued at the time stated, because the patient was to all appearance, and as far as she could discover, well; and because I was satisfied that the remains of the tumor would give no particular trouble, and would most likely disappear entirely.

At this date (April 20th, '78) Mrs. L. expresses herself as feeling better than she has for years. She has menstruated with tolerable regularity up to this time.

M. G. Sloan, M. D.

Charlotte, Iowa.

